# Genetic diversity of common *Gasterophilus* spp. from distinct habitats in China

**DOI:** 10.1186/s13071-018-3042-y

**Published:** 2018-08-22

**Authors:** Boru Zhang, Heqing Huang, Haoyu Wang, Dong Zhang, Hongjun Chu, Xinping Ma, Yan Ge, Make Ente, Kai Li

**Affiliations:** 1 Key Laboratory of Non-Invasive Research Technology for Endangered Species, College of Nature Conservation, Beijing Forestry University, Beijing, 100083 China; 2 Qinhuangdao Forestry Bureau, Qinhuangdao, 066004 Hebei China; 3 Wildlife Conservation Office of Altay Prefecture, Altay, 836599 Xinjiang China; 4 Xinjiang Research Centre for Breeding Przewalski’s Horse, Urumqi, 831700 Xinjiang China

**Keywords:** *Gasterophilus* spp., Mitochondrial DNA, Genetic diversity, Population genetic structure

## Abstract

**Background:**

*Gasterophilus* species are widely distributed around the world. The larvae of these flies parasitize the digestive tract of equids and cause damage, hindering horse breeding and protection of endangered species. However, study of the genetic structure of geographically distinct *Gasterophilus* populations is lacking. Here, we analyzed the genetic diversity of *Gasterophilus pecorum*, *G. intestinalis*, *G. nasalis* and *G. nigricornis* from three typical grasslands (meadow, desert and alpine steppes) in China as compared to published sequences from Italy, Poland and China (Daqing and Yili), based on the mitochondrial cytochrome *c* oxidase *cox*1 and *cox*2 gene sequences.

**Results:**

Haplotype diversity and nucleotide diversity of mitochondrial genes was generally high in all *Gasterophilus* populations. Due to the unique natural climatic conditions of the alpine steppe, there were high levels of genetic differentiation among different geographical populations of *G. pecorum* and *G. nasalis*, indicating that environmental variations influenced population genetic structure. Frequent exchanges between meadow and desert steppe *Gasterophilus* species resulted in low genetic differentiation. The highest exchange rates were found among *G. intestinalis* populations. Genetic differentiation was only observed on a large geographical scale, which was confirmed by analyzing population genetic structure. Three species, *G. pecorum*, *G. intestinalis* and *G. nasalis*, from meadow steppe showed a high emigration rate, indicating that the direction of *Gasterophilus* dispersal in China was from east to west.

**Conclusions:**

Our results show that the four *Gasterophilus* species have a high level of genetic diversity and different degrees of genetic differentiation and gene flow among different populations of the same species, reflecting their potential to adapt to the environment and the environmental impact on genetic structure. Knowledge of the genetic structure, population history, and migration will help understand the occurrence and prevalence of gasterophilosis and provide a basis for controlling the local spread of *Gasterophilus* spp.

**Electronic supplementary material:**

The online version of this article (10.1186/s13071-018-3042-y) contains supplementary material, which is available to authorized users.

## Background

*Gasterophilus* species are common obligate parasites in equines and are widely distributed worldwide. The genus *Gasterophilus* (Diptera: Oestridae) includes nine species [1]; of these, six are found in China: *G. pecorum*, *G. intestinalis*, *G. nasalis*, *G. nigricornis*, *G. haemorrhoidalis* and *G. inermis* [2–4]. *Gasterophilus* larvae parasitize the gastrointestinal tract of equids for 10–11 months and cause mucosal lesions, gastrointestinal ulcers, peritonitis, anemia and gastric rupture, which can be severely debilitating [5, 6]. The adults lay eggs directly on the hairs of host’s body, except *G. pecorum*, which lays its eggs on grass [1, 7]. In European and American countries where animal husbandry is highly developed, the most prevalent species are *G. intestinalis* and *G. nasalis*, with the other *Gasterophilus* species rarely observed [6–13]. In traditional Chinese pastoral areas (i.e. Inner Mongolia, Xinjiang and Qinghai) the distribution of *Gasterophilus* larvae is more complicated than in other regions. For instance, Xilin Gol Grassland of Inner Mongolia Autonomous Region is an important pastoral area in China with an abundance of grass and water. Six species of *Gasterophilus* were found in Inner Mongolia, the predominant species being *G. intestinalis* followed by *G. nasalis*, which is consistent with the prevalence of *Gasterophilus* in other countries and regions [14]. The diversity index of *Gasterophilus* in this region was found to be 1.31 [14]. The same *Gasterophilus* species have been found in Xinjiang and Inner Mongolia, but *G. pecorum* was the predominant species (96.17%) in Xinjiang, where the *Gasterophilus* species diversity index was very low (0.21) [14]. In Maduo County (MD) of Qinghai Province, only *G. pecorum* and *G. nasalis* have been documented, with the former being the predominant species (93.71%) and contributing to a low diversity index (0.23) [14].

Mitochondrial DNA markers have been widely used in studies of insect taxonomy, population genetics and evolution. More specifically, mitochondrial cytochrome *c* oxidase *cox*1 and *cox*2 genes have been used to examine inter- and intraspecific relationships in Diptera, Lepidoptera, Coleoptera, Hymenoptera and Hemiptera [15–19]. Previous investigations based on partial mitochondrial *cox*1 gene sequences revealed a high degree of genetic diversity that enabled differentiation of the Italian and Polish populations by PCR-restriction fragment length polymorphism [20]. In *G. intestinalis* from Daqing (DQ) and Yili (YL), China, genetic information is available for three mitochondrial genes, including *cox*1, NADH dehydrogenase subunit 5 gene (*nad*5) and *23S* ribosomal RNA gene [21]. However, little is known about the genetic structure of geographically distinct *Gasterophilus* populations. In the present study, we explored the population genetic structure and haplotype distribution patterns in several populations of *G. pecorum*, *G. intestinalis*, *G. nasalis* and *G. nigricornis* in China based on analyses of mitochondrial *cox*1 and *cox*2 gene sequences.

## Methods

### Study sites and sample collection

From February 2014 to December 2015, third-stage *Gasterophilus* larvae were collected in Kalamaili Nature Reserve (KNR) of Xinjiang Uigur Autonomous Region, Duolun County (DL) of Inner Mongolia Autonomous Region and MD of Qinghai Province (Additional file 1: Figure S1). A total of 97 *G. pecorum* and 63 *G. nasalis* specimens were obtained from the three locations, 42 *G. intestinalis* specimens were obtained from KNR and DL and 45 *G. nigricornis* specimens were obtained from KNR and DL (Additional file 2: Table S1). Larvae were preserved in ethanol and identified based on morphology [1].

Among the sampling sites in this study, KNR of Xinjiang Uigur Autonomous Region is located in the desert subregion of northwestern China with an altitude of 600–1464 m, average annual temperature of 2.4 °C, average summer temperature of 22 °C, average annual precipitation of 159 mm and annual evaporation of 2090 mm [22]. DL is located at the southern end of Xilin Gol Grassland in Inner Mongolia and is part of the Eurasian steppe; it has an elevation of 1150–1800 m, average annual temperature of 1.6 °C, average summer temperature of 18.2 °C, average annual precipitation of 385 mm and average annual evaporation of 1449.4–1672.8 mm [23]. Xinjiang and Inner Mongolia are zoogeographical regions belonging to the same district in China [24, 25]. MD is located in southwest Qinghai Province northwest of Guoluo Tibetan Autonomous Prefecture and has a plateau continental climate, the altitude is 4500–5000 m, annual average temperature is -4.1 °C, average summer temperature is < 10 °C, average annual precipitation is 303.9 mm, and average annual evaporation is 1331.21 mm [26].

### DNA extraction, amplification and sequencing

Total genomic DNA was extracted using a standard phenol/chloroform protocol [27], with minor modifications. Extracted DNA was frozen in DNase-free water and stored at -20 °C. A 632 bp fragment of the mitochondrial *cox*1 gene was amplified with primers Gco1s (5'-CAG TTG GAA TAG ACG TTG ATA CTC-3') and Gco1an (5'-AGG AAG TTC AGA ATA GCA GTG TTC-3') [20], and a 688 bp fragment of the mitochondrial *cox*2 gene was amplified with primers TL2-J-3037 (5'-ATG GCA GAT TAG TGC AAT GG-3') and TK-N-3785 (5'-GTT TAA GAG ACC AGT ACT TG-3') [28]. PCR amplification of *cox*1 was performed according to a previously described protocol [20]. PCR conditions for *cox*2 were as follows: 94 °C for 2 min, 35 cycles of 94 °C for 30 s, 55 °C for 30 s, and 72 °C for 1 min, and 25 °C for 2 min. PCR products were visualized by using 1% agarose gel electrophoresis, purified with the BigDye XTerminator Purification kit (Applied Biosystems, Foster City, CA, USA) and sequenced on an ABI 3730XL Genetic Analyzer (Applied Biosystems, Foster City, CA, USA). Sequences obtained were deposited in the GenBank database under the accession numbers MG815141-MG815634.

### Data analysis

Sequences generated in this study as well as published sequences of *G. intestinalis* (GenBank: GU265738-GU265748, GU299281-GU299283 and KR230402–KR230415) and *G. nasalis* (GenBank: GU265749-GU265758 and GU299284-GU299286) were used for data analysis. Raw sequences were proofread and edited using BioEdit v.7.0.9.0 [29]. Alignment was performed with the ClustalW algorithm in MEGA v.7.0 software [30], which was also used for basic sequence statistical analysis. Haplotype identification, haplotype diversity (*Hd*), and nucleotide diversity (*π*) were estimated using DnaSP v.5.0 [31]. The Kimura two-parameter method was used to calculate genetic distances among populations in MEGA. DnaSP v.5.0 was used to evaluate population pairwise fixation index (*Fst*) statistics and gene flow (*Nm*) values. Phylogenetic trees were constructed by the neighbor-joining (NJ) method using MEGA and were assessed with 1000 bootstrap replicates [32]. Haplotype networks were constructed using TCS v.1.21 [33] with a 95% parsimony criterion. The Likelihood Analysis with Metropolis Algorithm using Random Coalescence (LAMARC) programme [34] was used to estimate population size (*θ*), exponential growth rate (*g*) and migration rate based on the maximum-likelihood approach. The search strategy included three replications of 10 short initial chains and two long final chains. The initial chains were run with 500 samples and the sampling interval was set to 20. The final chains were performed with 10,000 samples with the same sampling interval. A burn-in of 1000 samples was used for each chain.

## Results

### Haplotype diversity

A total of 247 *cox*1 sequences were obtained for *G. pecorum*, *G. intestinalis*, *G. nasalis* and *G. nigricornis*, co-analyzed with 41 published sequences of *G. intestinalis* from China (DQ and YL), Italy and Poland, and of *G. nasalis* from Italy and Poland. An analysis of all *cox*1 sequences identified a total of 154 haplotypes. The haplotype distribution, *Hd* and *π* of each population based on the mitochondrial *cox*1 gene are shown in Additional file 2: Table S1. The values for *Hd* and *π* were as follows: *G. pecorum* (*Hd* = 0.976, *π* = 0.01581); *G. intestinalis* (*Hd* = 0.983, *π* = 0.01066); *G. nasalis* (*Hd* = 0.953, *π* = 0.01226); and *G. nigricornis* (*Hd* = 0.973, *π* = 0.01719). For each species, only two to five common haplotypes were observed within localities in China and there were no shared haplotypes across *G. intestinalis* and *G. nasalis* populations in Italy and Poland.

The 247 *cox*2 sequences of the four *Gasterophilus* species defined 127 haplotypes. The haplotype distribution, *Hd* and *π* of each regional population based on the mitochondrial *cox*2 gene are shown in Additional file 2: Table S2. The values for *Hd* and *π* were as follows: *G. pecorum* (*Hd* = 0.01093, *π* = 0.959); *G. intestinalis* (*Hd* = 0.00592, *π* = 0.951); *G. nasalis* (*Hd* = 0.01003, *π* = 0.961); and *G. nigricornis* (*Hd* = 0.01217, *π* = 0.918). Similar to *cox*1, only two to six common *cox*2 haplotypes were observed within populations of each species.

### Haplotype networks

#### *Gasterophilus pecorum*

**Fig. 1:**
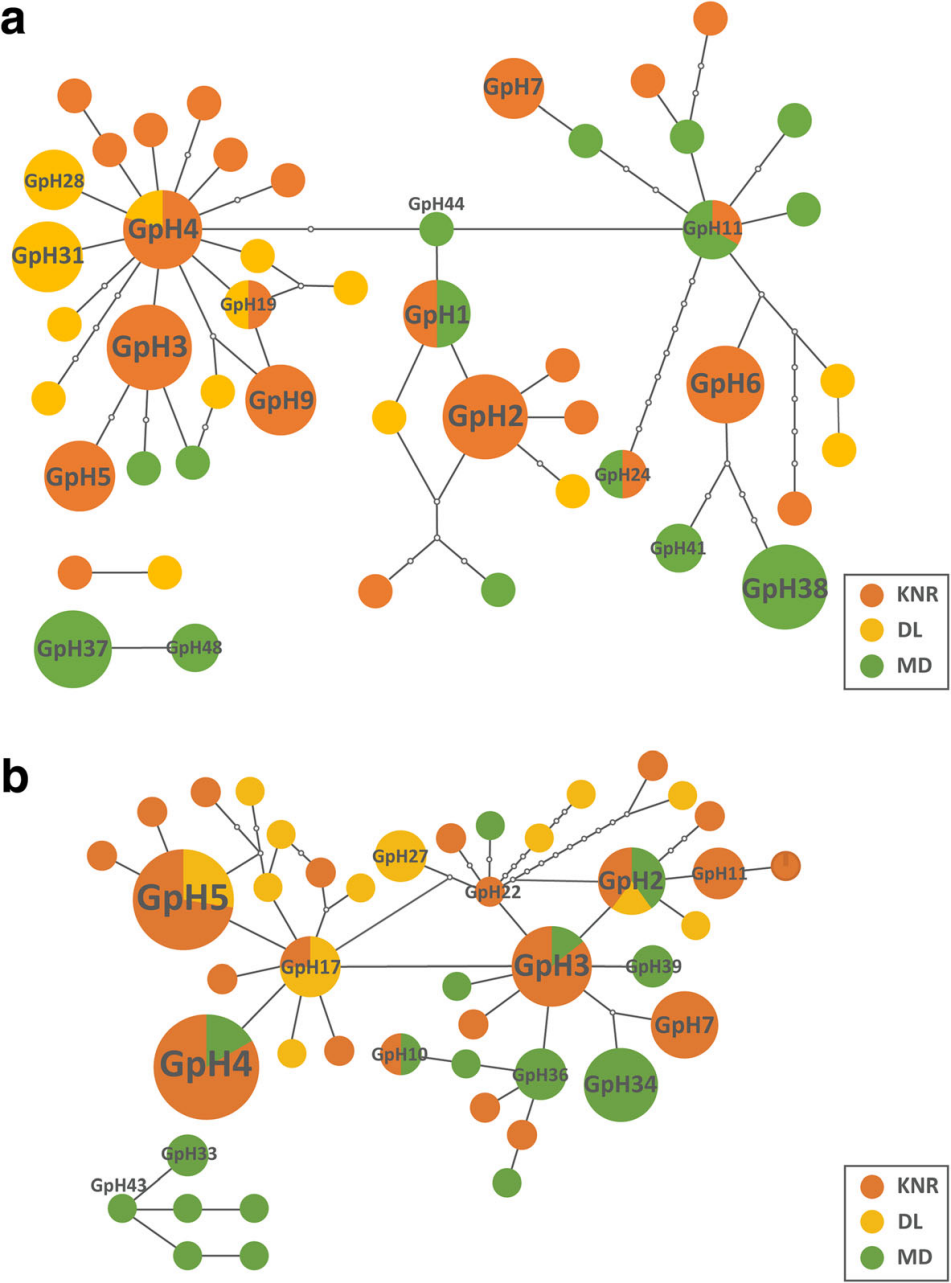
Haplotype networks for *G. pecorum*. **a** Haplotype network for *G. pecorum* based on the *cox*1 gene. **b** Haplotype network for *G. pecorum* based on the *cox*2 gene

The minimum spanning network calculated with TCS software using *cox*1 haplotypes of *G. pecorum* had three sub-networks (Fig. 1a). Of the 48 *cox*1 haplotypes, two (GpH4 and GpH19) were shared between KNR and DL and three (GpH1, GpH11 and GpH24) were shared between KNR and MD, while no common haplotypes were detected between DL and MD. The sub-networks formed a star-like structure that was derived from haplotype GpH4. Most unique haplotypes in the DL population were distributed around GpH4, whereas those unique to the MD population mostly surrounded GPH11. Haplotypes in the KNR population were more evenly distributed throughout the network. Individuals of GpH37 and GpH48 constituting a single sub-network were all from MD. In general, the *cox*2 haplotypes formed two sub-networks with GpH17 and GpH43 as the central haplotypes (Fig. 1b). The haplotype distribution of each geographical population was similar to that of *cox*1. Individuals in the GpH43-centric sub-network were the same as those in the *cox*1 haplotype sub-network comprising only MD haplotypes. Of the 45 *cox*2 haplotypes, three (GpH2, GpH5 and GpH17) were shared between KNR and DL, four (GpH2, GpH3, GpH4 and GpH10) were shared between KNR and MD, and GpH2 was shared by all three populations.

#### *Gasterophilus intestinalis*

**Fig. 2:**
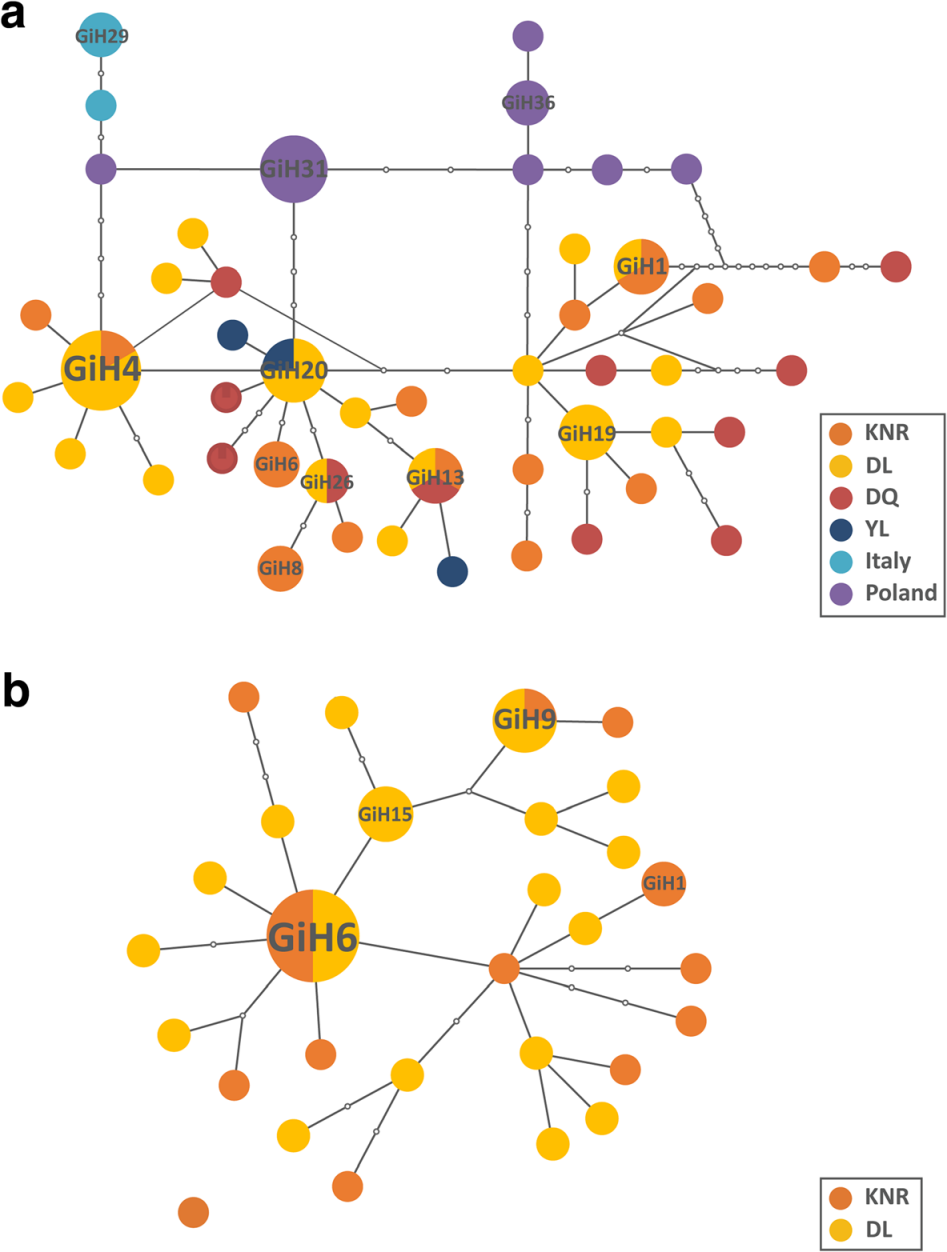
Haplotype networks for *G. intestinalis*. **a** Haplotype network for *G. intestinalis* based on the *cox*1 gene. **b** Haplotype network for *G. intestinalis* based on the *cox*2 gene

The minimum spanning network calculated with TCS software using *cox*1 haplotypes of *G. intestinalis* had a single network and no sub-networks. Of the 48 *cox*1 haplotypes, three (GiH1, GiH4 and GiH13) were shared by KNR and DL, and one each was shared by KNR and DQ (GiH13), DL and DQ (GiH26), and DL and YL (GiH20). The network structure was dominated by a single haplotype (GiH4) (Fig. 2a). Haplotypes from Italy and Poland were all private haplotypes (i.e. found in only one population) and were less directly linked to those from China localities. The *cox*2 haplotype network had a star-like structure, with the most abundant haplotype (GiH6) in the center and distributed across all geographical regions (KNR and DL) (Fig. 2b). The second most frequent haplotype GiH9 was also shared by KNR and DL.

#### *Gasterophilus nasalis*

**Fig. 3:**
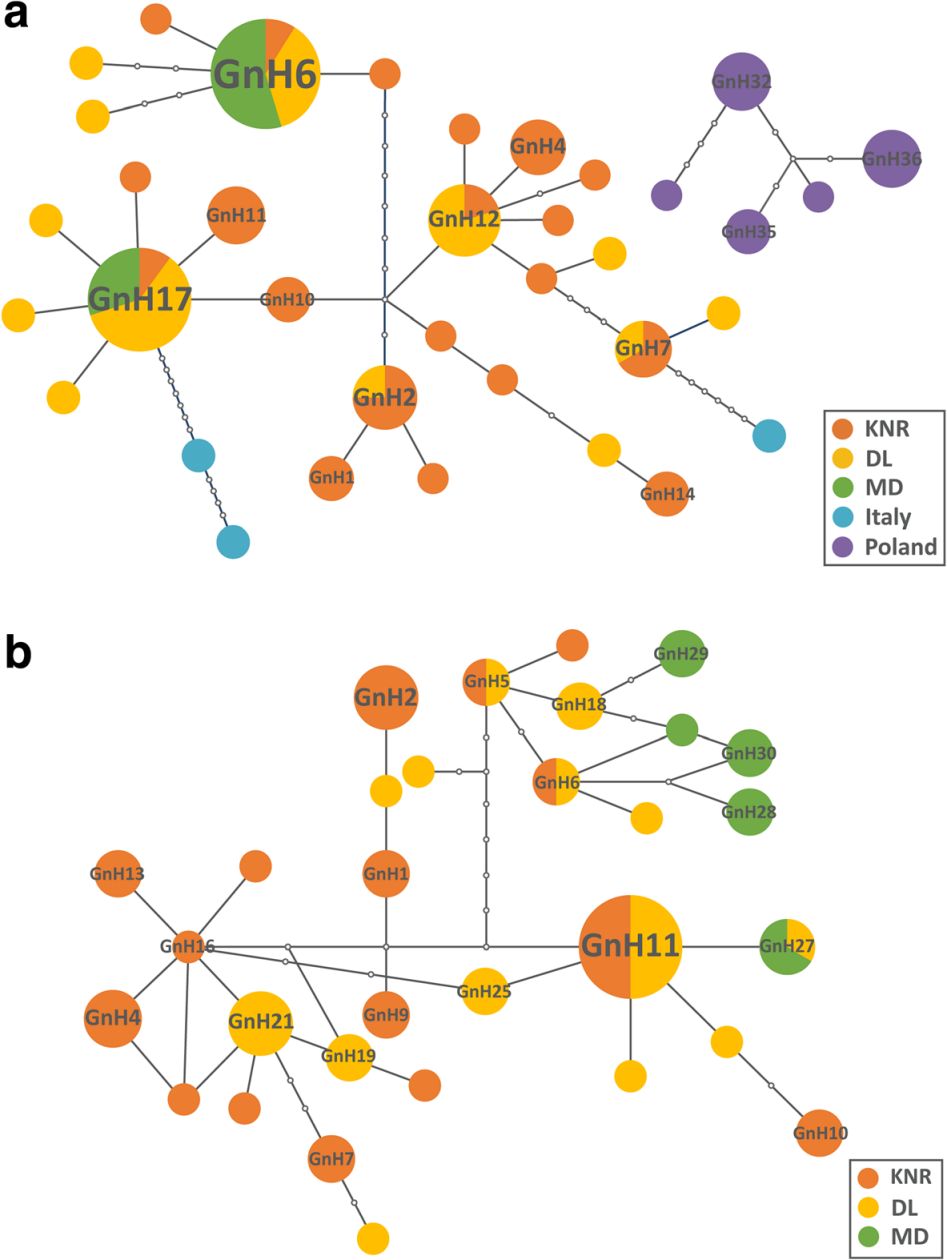
Haplotype networks for *G. nasalis*. **a** Haplotype network for *G. nasalis* based on the *cox*1 gene. **b** Haplotype network for *G. nasalis* based on the *cox*2 gene

The minimum spanning network calculated with TCS software using *cox*1 haplotypes of *G. nasalis* had two sub-networks. Four (GnH2, GnH6, GnH7 and GnH17) of the 33 haplotypes were shared between KNR and DL, and two (GnH6 and GnH17) were shared among KNR, DL and MD (Fig. 3a). All haplotypes from Italy and Poland were private. Haplotypes in Poland constituted a separate sub-network, while those in Italy were less directly linked to haplotypes from localities in China. The *cox*2 haplotype network was dominated by GnH16, which was represented by a single individual (Fig. 3b). Haplotypes GnH5, GnH6 and GnH11 were shared between KNR and MD, while GnH27 was common to DL and MD. The MD haplotypes were mostly focused on branches centered on GnH5, there was no similar phenomenon in the *cox*1 haplotype network. Haplotypes in KNR and DL were randomly distributed throughout the network.

#### *Gasterophilus nigricornis*

**Fig. 4:**
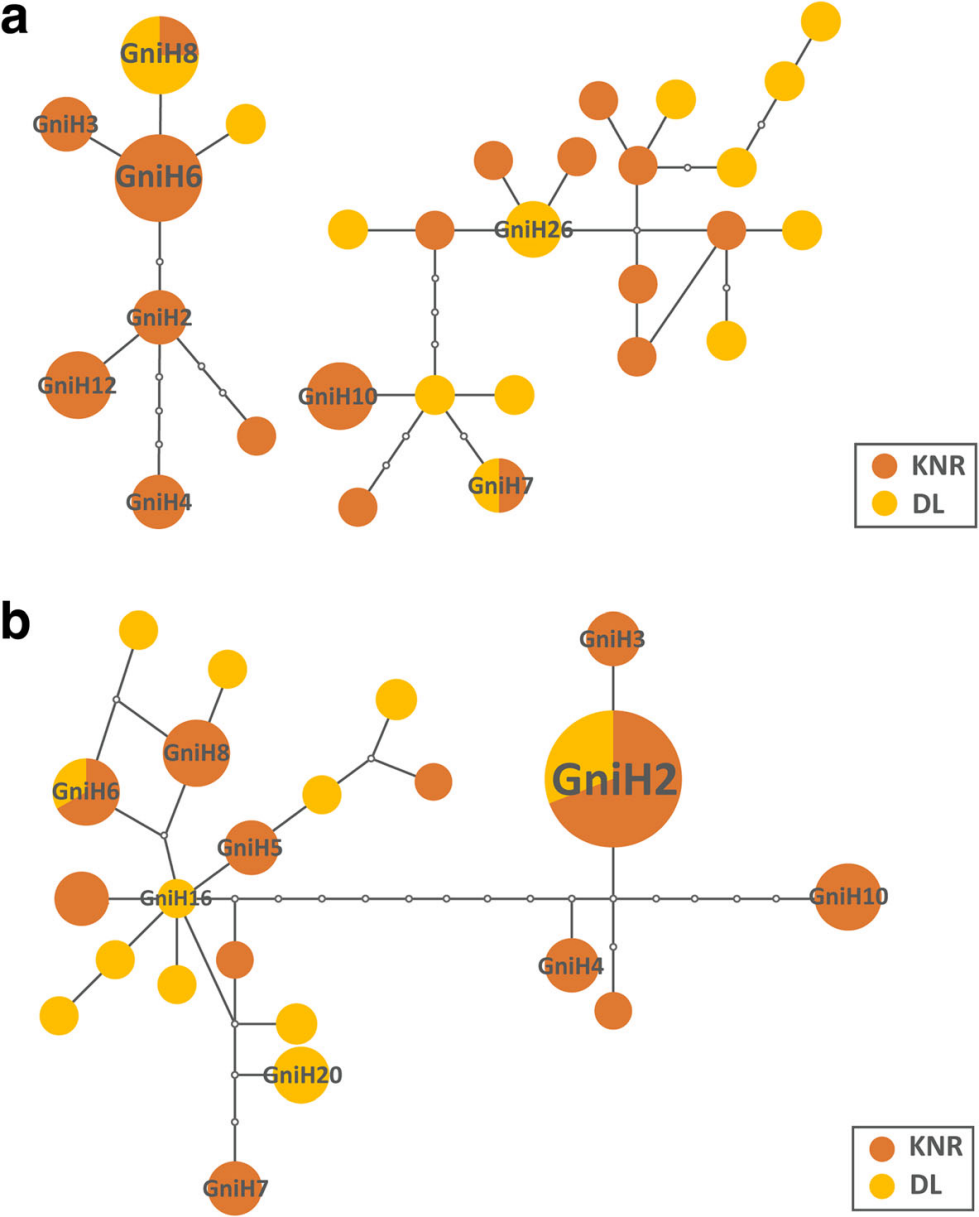
Haplotype networks for *G. nigricornis*. **a** Haplotype network for *G. nigricornis* based on the *cox*1 gene. **b** Haplotype network for *G. nigricornis* based on the *cox*2 gene

The minimum spanning network calculated with TCS software using *cox*1 haplotypes of *G. nigricornis* had two sub-networks centered around GniH6 and GniH26 (Fig. 4a). Most GL haplotypes were distributed around GniH26. The *cox*2 haplotype network comprised a single network centered around haplotype GniH16 (Fig. 4b). Five of the 22 haplotypes differed by more than eight mutations from haplotype GniH16, and most DL haplotypes were more closely related to the ancestral one.

### Phylogenetic analysis

#### *Gasterophilus pecorum*

**Fig. 5:**
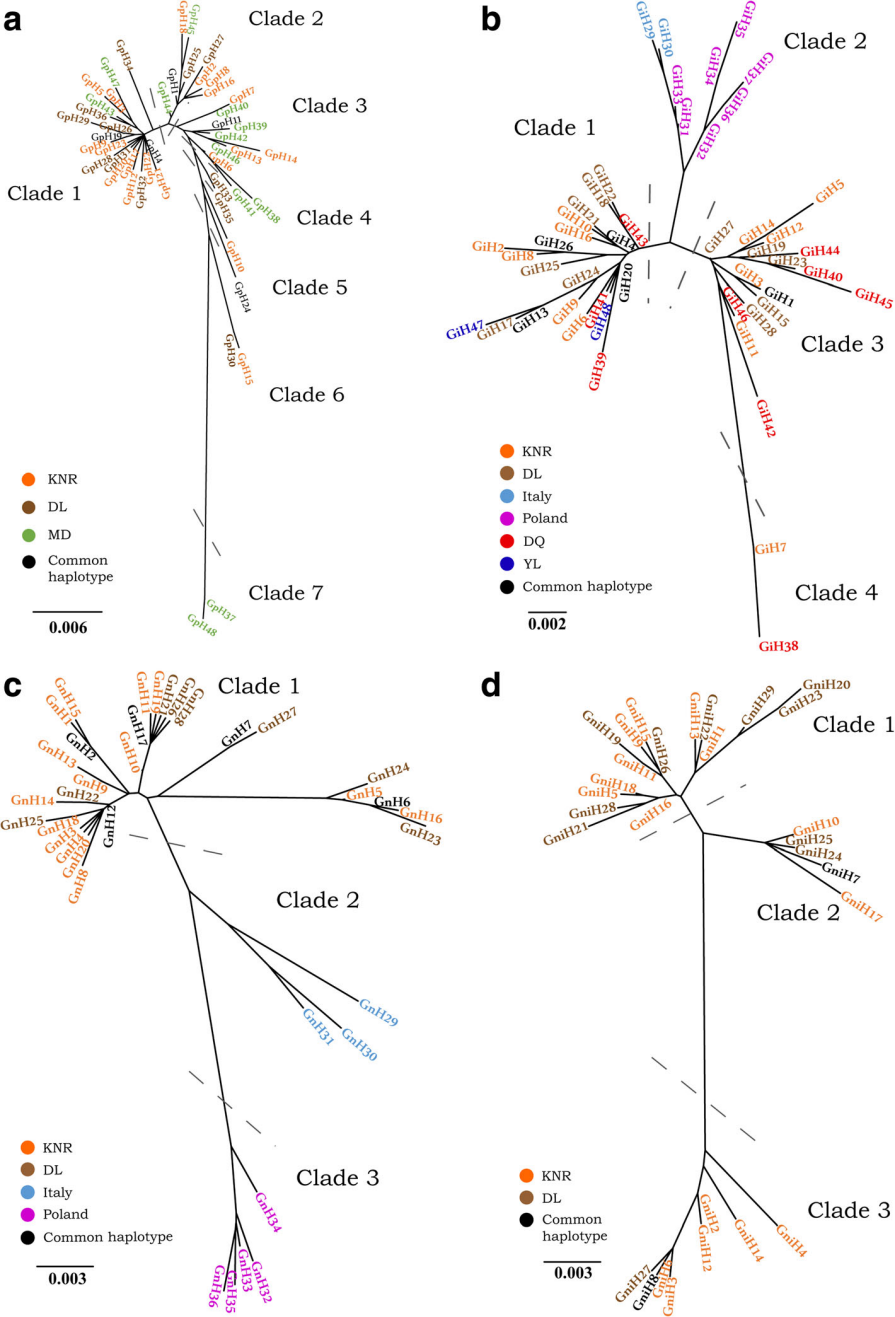
Neighbor-joining tree based on the *cox*1 gene for *G. pecorum*, *G. intestinalis*, *G. nasalis* and *G. nigricornis*. **a** *Gasterophilus pecorum*. **b** *Gasterophilus intestinalis*. **c** *Gasterophilus nasalis*. **d** *Gasterophilus nigricornis*

**Fig. 6:**
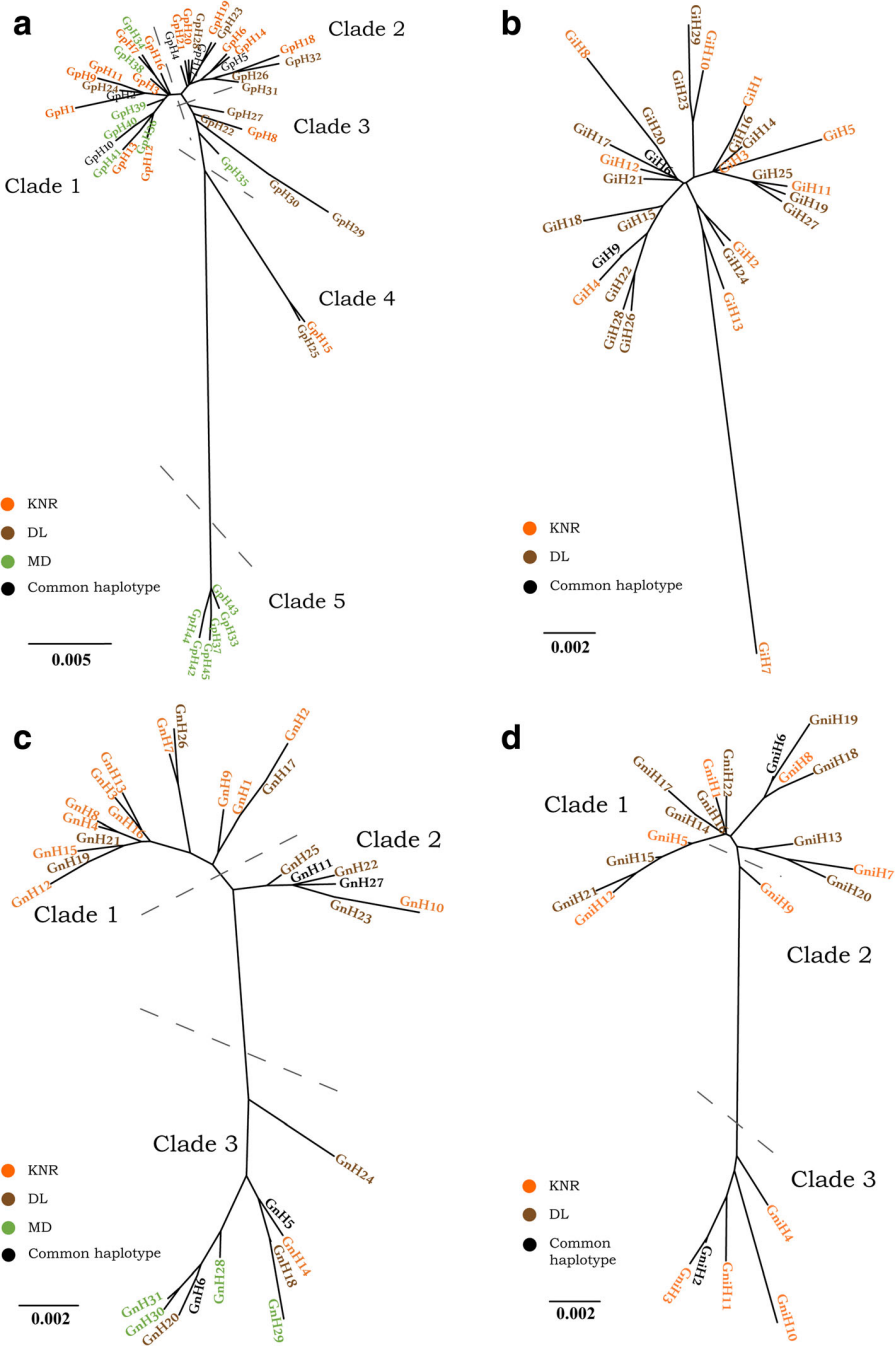
Neighbor-joining tree based on the *COII* gene for *G. pecorum*, *G. intestinalis*, *G. nasalis* and *G. nigricornis*. **a** *Gasterophilus pecorum*. **b** *Gasterophilus intestinalis*. **c** *Gasterophilus nasalis*. **d** *Gasterophilus nigricornis*

The phylogenetic tree constructed with the NJ method based on the *cox*1 gene included 48 haplotypes divided into seven clades (Fig. 5a). Clade 1 included 73.68% of DL and 50% of KNR samples with seven and nine private haplotypes, respectively. Clades 3 and 7 comprised 77.42% of MD samples, and all haplotypes except GpH41 and GpH44 were private. An NJ tree constructed from *cox*2 sequences had five clades (Fig. 6a). DL haplotypes, including nine that were private, were mainly distributed in Clades 2 and 3, accounting for 84.21% of individuals in the DL population. Clades 1 and 5 contained the remaining 13 private haplotypes of MD except for GpH35, and included 87.10% of individuals from MD. KNR haplotypes were mainly found in Clades 1 and 2, the former included seven private haplotypes and 40% of individuals, while the latter contained six private haplotypes and 50% of individuals.

#### *Gasterophilus intestinalis*

The NJ tree constructed based on the *cox*1 gene included 44 haplotypes that were divided into four clades (Fig. 5b). Haplotypes from Italy and Poland were independently clustered and all were distributed in Clade 2, with the populations from the four geographical regions of China forming the three remaining clades. In the NJ tree constructed based on *cox*1 haplotypes, KNR and DL populations did not show any obvious clustering (Fig. 6b).

#### *Gasterophilus nasalis*

The NJ tree constructed based on *cox*1 haplotypes had three clades (Fig. 5c). Three of the Chinese populations (KNR, DL and MD) formed Clade 1, and the Italian and Polish populations formed Clades 2 and 3, respectively. The NJ tree constructed based on the *cox*2 gene had 31 haplotypes divided into three clades (Fig. 6c). The KNR population was mainly distributed in Clade 1, which included 10 private haplotypes and 66.67% of KNR individuals. All four private haplotypes of MD were distributed in clade 3, which included 77.78% of MD individuals. The DL population was distributed in three clades with no obvious aggregation.

#### *Gasterophilus nigricornis*

The NJ tree constructed from *cox*1 haplotypes was divided into three clades (Fig. 5d). The DL population was mainly distributed in Clade 1, which contained eight private haplotypes and 56.25% of DL individuals, whereas the KNR population was evenly distributed. The NJ tree constructed based on *cox*1 haplotypes was also divided into three clades (Fig. 6d). The private haplotypes of DL were all distributed in Clade 1, which included 68.75% of DL individuals. The haplogroup clustering of the KNR population was similar to that of *cox*1.

### Genetic structure and gene flow

#### *Gasterophilus pecorum*

Mean genetic distances between different populations were calculated based on *cox*1 and *cox*2 sequences. Distances based on *cox*1 gene sequences among DL, KNR and MD populations were 0.008 (KNR and DL), 0.023 (KNR and MD) and 0.024 (DL and MD), whereas distances based on *cox*2 gene sequences were 0.006 (KNR and DL), 0.015 (KNR and MD) and 0.017 (DL and MD). These results demonstrate that the genetic distance was smallest between KNR and DL populations and larger than for other populations. *Fst* values for *cox*1 sequences were 0.03245 (KNR and DL), 0.19067 (KNR and MD) and 0.22841 (DL and MD), while the corresponding *Nm* values were 7.45416, 1.06116 and 0.84452, respectively. *Fst* values for *cox*2 sequences were 0.04216 (KNR and DL), 0.17652 (KNR and MD) and 0.17893 (DL and MD), and *Nm* values were 5.67979, 1.16627 and 1.14719, respectively. These results indicate that there was a low degree of genetic differentiation (*Fst* < 0.05, *Nm* > 4) between KNR and DL populations, and moderate genetic differentiation (0.15 < *Fst* < 0.25) and low level of gene flow (*Nm* < 4) between MD and the other two populations.

#### *Gasterophilus intestinalis*

Genetic distances among different populations based on *cox*1 sequences are shown in Table 1. Mean genetic distances among Chinese, Italian and Polish populations ranged between 0.018–0.023. The distances among populations in China did not differ significantly (0.009–0.012), whereas the distance between KNR and DL populations based on *cox*2 sequences was 0.006. *Fst* and *Nm* values for *cox*1 sequences among the six populations are shown in Table 2. Considerable genetic differentiation (*Fst* > 0.25) and low level of gene flow (*Nm* < 4) occur among Italian, Polish and Chinese populations, but restricted by the very small sample size of Italian population, this result remains to be verified. Genetic differentiation was low among KNR, DL and DQ populations (*Fst* < 0.05, *Nm* > 4) and moderate among the remaining populations in China (0.05 < *Fst* < 0.15), which also exhibited a low degree of gene flow (*Nm* < 4). *Fst* and *Nm* values for *cox*2 sequences between KNR and DL were 0.01847 and 13.28546, respectively, indicating a low level of genetic differentiation (*Fst* < 0.05) and frequent gene flow (*Nm* > 4).

**Table 1:** Mean genetic distance between *G. intestinalis* populations in distinct areas based on mitochondrial cytochrome *c* oxidase subunit 1 gene

Population	KNR	DL	Italy	Poland	DQ
DL	0.009	–	–	–	–
Italy	0.018	0.015	–	–	–
Poland	0.014	0.013	0.009	–	–
DQ	0.012	0.010	0.019	0.016	–
YL	0.010	0.007	0.014	0.013	0.012

**Table 2:** Pairwise fixation index (*Fst*: below diagonal) and gene flow (*Nm*: above diagonal) values for six *G. intestinalis* populations

Population	KNR	DL	Italy	Poland	DQ	YL
KNR	–	9.42867	0.14350	0.32216	-12.92101	1.88931
DL	0.02583	–	0.10156	0.22386	13.38884	2.84521
Italy	0.63532	0.71112	–	0.18810	0.17816	0.11111
Poland	0.43694	0.52758	0.57065	–	0.39733	0.20365
DQ	-0.01973	0.01833	0.58389	0.38620	–	1.70542
YL	0.11686	0.08077	0.69231	0.55109	0.12785	–

#### *Gasterophilus nasalis*

Mean genetic distances among populations based on *cox*1 sequences are shown in Table 3. Distances based on *cox*2 sequences were 0.009 (KNR and DL), 0.015 (KNR and MD) and 0.013 (DL and MD), and those based on *cox*1 sequences ranged between 0.009–0.023, with the closest genetic distance observed between KNR and DL populations. The sequence dataset for *cox*2 also revealed a close genetic distance between KNR and DL and showed that MD was distant from the other populations. *Fst* and *Nm* values for the *cox*1 gene among the four populations are shown in Table 4. Apart from the low degree of genetic differentiation (*Fst* < 0.05) and frequent gene flow (*Nm* > 4) between KNR and DL, the other populations showed high genetic differentiation (*Fst* > 0.25) and low gene flow (*Nm* < 4).

**Table 3:** Mean genetic distance between *G. nasalis* populations in distinct areas based on mitochondrial cytochrome *c* oxidase subunit 1 gene

Population	KNR	DL	MD	Italy
DL	0.009	–	–	–
MD	0.012	0.011	–	–
Italy	0.020	0.021	0.023	–
Poland	0.018	0.019	0.023	0.017

**Table 4:** Pairwise fixation index (*Fst*: below diagonal) and gene flow (*Nm*: above diagonal) values for five *G. nasalis* populations

Population	KNR	DL	MD	Italy	Poland
KNR	–	9.00583	0.51422	0.26556	0.16560
DL	0.02701	–	1.27588	0.29397	0.17310
MD	0.32713	0.16384	–	0.20223	0.11048
Italy	0.48491	0.45958	0.55282	–	0.29553
Poland	0.60015	0.59088	0.69352	0.45827	–

#### *Gasterophilus nigricornis*

The mean genetic distances between KNR and DL populations calculated based on *cox*1 and *cox*2 sequences were 0.019 and 0.013, respectively. *Fst* and *Nm* values for the *cox*1 gene between KNR and DL were 0.07371 and 3.14167, respectively, while those for the *cox*2 gene were 0.08215 and 2.79321, respectively. These results showed that there was little genetic differentiation (*Fst* > 0.05) and low level of gene flow (*Nm* < 4) between KNR and DL.

### Demographic history and migration

Populations are often far from equilibrium, and not all deviate in the same direction. The neutrality of all populations was estimated using Tajima’s *D* and Fu’s *Fs* (Additional file 2: Table S3). The results of the neutrality test indicated a recent rapid expansion in two populations (KNR and DL) of *G. pecorum*, three populations (KNR, DL and DQ) of *G. intestinalis*, and in the KNR population of *G. nasalis*, whereas the other populations had remained relatively stable.

The LAMARC analysis (Additional file 2: Table S4) revealed that population size (*θ*) and exponential growth rate (*g*) were relatively high for the DL population followed by the KNR population of *G. pecorum*, while the low *θ* value and negative *g* value of the MD population indicated a declining trend. Migration was high from DL to KNR and from KNR to MD, whereas the emigration and immigration rates of MD and DL, respectively, were low. Among *G. intestinalis* populations, DQ had the highest *θ* and *g* values followed by KNR. High migration was observed among Chinese populations, including from DL to KNR, DL to YL, DL to DQ and YL to DQ. In general, KNR populations had low emigration and high immigration rates, while the opposite was true for DL. In European populations, migration was more frequent from Poland to Italy than in the other direction. The relationship between the Italian and Chinese populations was more complex than that between the Polish and Chinese populations, but the results may be biased due to the limited number of samples from Italy. Among *G. nasalis*, the highest *g* and *θ* values were observed in the MD and Italy populations, respectively. In China, the highest rates of migration were from DL to KNR and MD. The migration rates from Italy to Poland were even higher, while those between the Chinese and European populations were generally low. The *θ* values of *G. nigricornis* in KNR and Dowered were similar, whereas *g* values were higher in DL than in KNR. Migration rates between the two populations were similarly high, with migration from KNR to DL being slightly higher.

## Discussion

In this study, analyses of the mitochondrial *cox*1 and *cox*2 genes of *G. pecorum*, *G. intestinalis*, *G. nasalis* and *G. nigricornis* revealed that besides *cox*1 gene haplotypes in the DL population of *G. nasalis*, the other populations of each species showed high *Hd*, indicating a high degree of *cox*1 and *cox*2 gene polymorphism in *Gasterophilus* species, all of which showed a high degree of concordance with fewer common haplotypes and fewer samples sharing the same haplotype. The high diversity of *G. pecorum* has been previously reported [35]. Compared to the mitochondrial *cox*1 gene, the *cox*2 sequences of *G. pecorum* and *G. nasalis* showed higher *Hd* and *π* and more private haplotypes. The haplotype networks and phylogenetic trees revealed that haplotypes of DL and MD populations of *G. pecorum* had distinct distributions that were similar to the trends of *G. nasalis*. The Italian, Polish and Chinese populations of *G. intestinalis* clustered into three clades, all Chinese isolates clustered together without geographical separation. Compared to the *G. nigricornis* KNR population, MD showed a certain degree of aggregation.

Host migration affects the genetic structure of parasite populations [36, 37]. The geographical distribution of species is closely related to biological and non-biological factors in the ecological niche, including exogenous factors such as geological events, changes in climate and food distribution as well as intrinsic species characteristics such as behavioral sensitivity, food web specificity and diffusibility. These factors act either directly or indirectly on both the host and parasite, with more profound effects on the latter [38]. The life-cycle of *Gasterophilus* spp. consists of the obligate parasitic larval stage on the horse and the free-living adult stage, and is influenced by host movement and environment. The three sampling sites in China selected for this study represent different types of grassland with distinct climates. Only *G. pecorum* and *G. nasalis* were collected in MD, our analysis revealed a moderate degree of genetic differentiation and a lack of gene flow between MD and populations in other regions. In contrast, more frequent genetic flow was observed between the KNR and DL populations of all four species. In general, the four *Gasterophilus* species showed a consistent population genetic structure in the KNR and DL populations, whereas two of the species in MD differed from their counterparts in KNR and DL. This implies that even if there is host exchange as a part of livestock husbandry trade, the natural conditions of the Qinghai-Tibet Plateau characterized by an alpine climate and high altitude differ from those of the other two regions located on natural geographical barriers such as Kunlun and Qilian Mountains, which can lead to genetic differentiation while preventing gene exchange among populations. Xinjiang and Inner Mongolia constitute a single zoogeographical region (Mongo-Xinjiang region) [24, 25]; consistent with this, our results suggest that *Gasterophilus* populations in KNR of Xinjiang and DL of Inner Mongolia share similar a genetic structure and species type.

*Gasterophilus pecorum*, *G. intestinalis* and *G. nasalis* from DL showed a consistently high migration rate, suggesting that DL is the origin of *Gasterophilus* flies in Mongolia-Xinjiang and Qinghai-Tibet regions of China. The Xinjiang Uygur Autonomous Region includes KNR at the heart of the Eurasian continent. Historically, the Silk Road of China passed through this area, which saw frequent trade of animals such as horses that were important means of transportation [39]. The generally higher migration rates of *Gasterophilus* flies in KNR indicate that *Gasterophilus* spp. in the area spread from multiple locations, likely reflecting the frequent movement of their hosts. Although we sampled a limited number of sites in this study, our results nonetheless demonstrate that *Gasterophilus* spp. migrated westward from the grasslands of eastern China. The neutrality test results showed that *G. pecorum* and *G. intestinalis* underwent significant population expansion and were the predominant species in KNR and DL, respectively.

## Conclusions

We investigated the genetic structure and inter-population relationships of four common *Gasterophilus* species of different geographical populations based on analyses of mitochondrial *cox*1 and *cox*2 genes. Based on these results, we propose dispersal patterns of these species in China’s three major pastoral areas.

## Additional files

Additional file 1: **Figure S1.** Map of sampling sites in China. (PDF 5098 kb)

Additional file 2: **Table S1.** Number of individuals (*n*), haplotypes, number of haplotypes (denoted in parentheses), haplotype diversity (*Hd*), and nucleotide diversity (*π*) based on the mitochondrial cytochrome *c* oxidase subunit 1 gene in *G. pecorum*, *G. intestinalis*, *G. nasalis* and *G. nigricornis*. **Table S2.** Number of individuals (*n*), haplotypes, number of haplotypes (denoted in parentheses), haplotype diversity (*Hd*), and nucleotide diversity (*π*) based on the mitochondrial cytochrome *c* oxidase subunit 2 gene in *G. pecorum*, *G. intestinalis*, *G. nasalis* and *G. nigricornis*. **Table S3.** Neutrality test for *G. pecorum*, *G. intestinalis*, *G. nasalis* and *G. nigricornis* in different geographical locations. **Table S4.** Maximum likelihood estimates of population size (*θ*), exponential growth rate (*g*), and migration rate for different populations of *G. pecorum*, *G. intestinalis*, *G. nasalis* and *G. nigricornis*. (DOCX 27 kb)

## Abbreviations

*cox*1: Mitochondrial cytochrome *c* oxidase subunit 1 gene
*cox*2: Mitochondrial cytochrome *c* oxidase subunit 2 gene
DL: Duolun County, Inner Mongolia Autonomous Region
DQ: Daqing City, Heilongjiang Province
*Fst*: Pairwise fixation index
*g*: Exponential growth rate
*Hd*: Haplotype diversity
KNR: Kalamaili Nature Reserve, Xinjiang Uigur Autonomous Region
LAMARC: Likelihood Analysis with Metropolis Algorithm using Random Coalescence
MD: Maduo County, Qinghai Province
NJ: Neighbor-joining
*Nm*: Gene flow
YL: Yili Kazak Autonomous Prefecture, Xinjiang Uigur Autonomous Region
*θ*: Population size
*π*: Nucleotide diversity
