# Supplementary figures and images for: Genetic diversity of common Gasterophilus spp. from distinct habitats in China
Source: Parasit Vectors. 2018 Aug 22;11:474. doi: 10.1186/s13071-018-3042-y (PMC6106871; doi:10.1186/s13071-018-3042-y)

**Figure S1.** Map of sampling sites in China

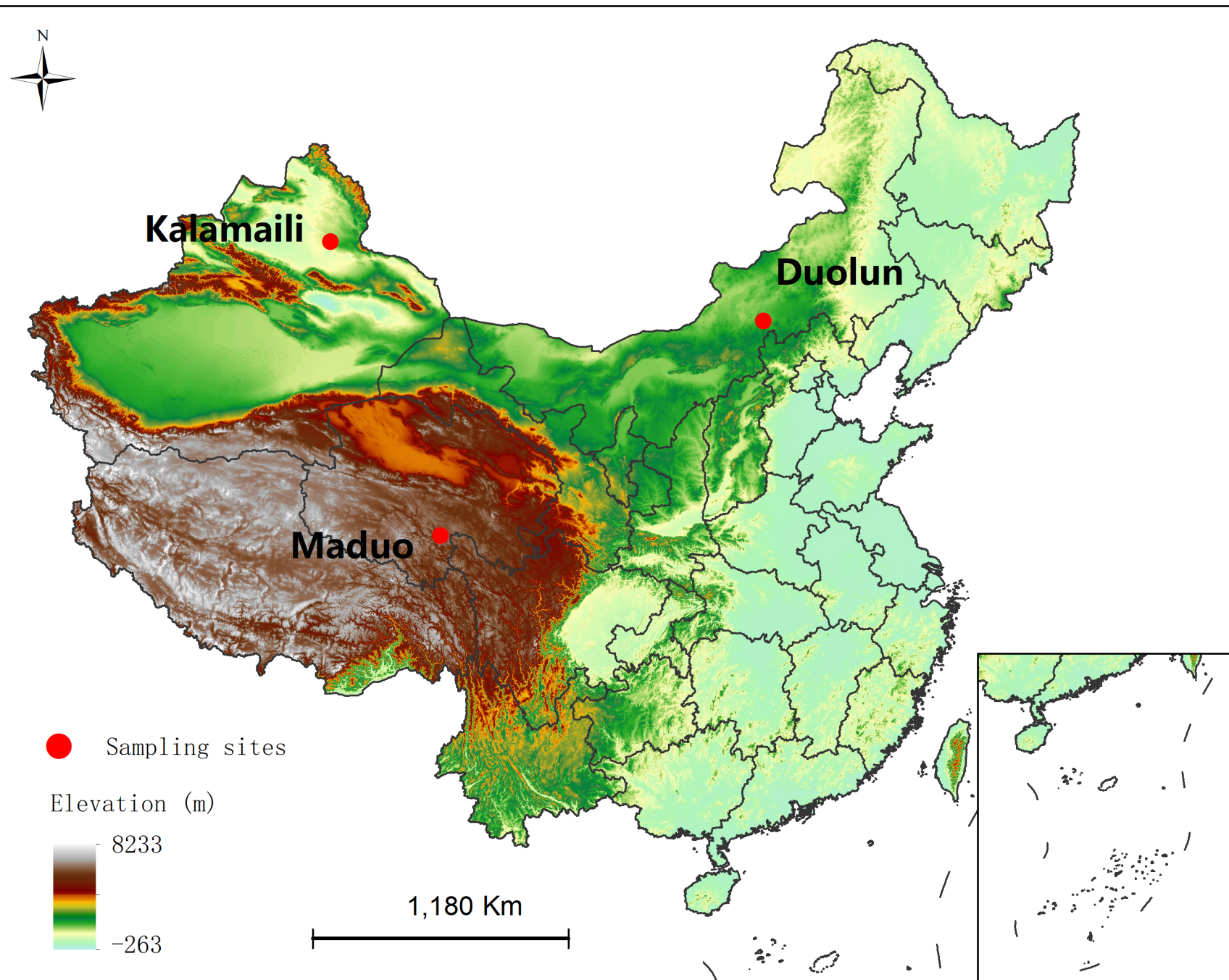

Supplement: Supplementary file 1 — Figure S1. Map of sampling sites in China. (PDF 5098 kb) [file 13071_2018_3042_MOESM1_ESM.pdf]
