# Supplementary material for: Genetic diversity of common Gasterophilus spp. from distinct habitats in China
Source: Parasit Vectors. 2018 Aug 22;11:474. doi: 10.1186/s13071-018-3042-y (PMC6106871; doi:10.1186/s13071-018-3042-y)
Supplement: Supplementary file 2 — Table S1. Number of individuals (n), haplotypes, number of haplotypes (denoted in parentheses), haplotype diversity (Hd), and nucleotide diversity (π) based on the mitochondrial cytochrome c oxidase subunit 1 gene in G. pecorum, G. intestinalis, G. nasalis and G. nigricornis. Table S2. Number of individuals (n), haplotypes, number of haplotypes (denoted in parentheses), haplotype diversity (Hd), and nucleotide diversity (π) based on the mitochondrial cytochrome c oxidase subunit 2 gene in G. pecorum, G. intestinalis, G. nasalis and G. nigricornis. Table S3. Neutrality test for G. pecorum, G. intestinalis, G. nasalis and G. nigricornis in different geographical locations. Table S4. Maximum likelihood estimates of population size (θ), exponential growth rate (g), and migration rate for different populations of G. pecorum, G. intestinalis, G. nasalis and G. nigricornis. (DOCX 27 kb) [file 13071_2018_3042_MOESM2_ESM.docx]

**Table S1.** Number of individuals (*n*), haplotypes, number of haplotypes (denoted in parentheses), haplotype diversity (*Hd*), and nucleotide diversity (*π*) based on the mitochondrial cytochrome oxidase I gene in *G. pecorum*, *G. intestinalis*, *G. nasalis*, and *G. nigricornis*.

| Species | Location | *n* | *COI* gene | | |
| --- | --- | --- | --- | --- | --- |
|  |  |  | *Hd* | *π* | Haplotypes |
| *G. pecorum* | KNR, China | 50 | 0.949 | 0.00815 | GpH1(2) GpH2(6) GpH3(6) GpH4(4) GpH5(4) GpH6(5) GpH7(3) GpH8(1) GpH9(4) GpH10(1) GpH11(1) GpH12(1) GpH13(1) GpH14(1) GpH15(1) GpH16(1) GpH17(1) GpH18(1) GpH19(1) GpH20(1) GpH21(1) GpH22(1) GpH23(1) GpH24(1) |
|  | DL, China | 19 | 0.947 | 0.00809 | GpH4(1) GpH19(1) GpH25(1) GpH26(1) GpH27(1) GpH28(3) GpH29(1) GpH30(1) GpH31(4) GpH32(1) GpH33(1) GpH34(1) GpH35(1) GpH36(1) |
|  | MD, China | 28 | 0.923 | 0.02787 | GpH1(2) GpH11(2) GpH24(1) GpH37(5) GpH38(6) GpH39(1) GpH40(1) GpH41(2) GpH42(1) GpH43(1) GpH44(1) GpH45(1) GpH46(1) GpH47(1) GpH48(2) |
| *G. intestinalis* | KNR, China | 17 | 0.978 | 0.01049 | GiH1(2) GiH2(1) GiH3(1) GiH4(1) GiH5(1) GiH6(2) GiH7(1) GiH8(2) GiH9(1) GiH10(1) GiH11(1) GiH12(1) GiH13(1) GiH14(1) |
|  | DL, China | 25 | 0.947 | 0.00628 | GiH1(1) GiH4(5) GiH13(1) GiH15(1) GiH16(1) GiH17(1) GiH18(1) GiH19(3) GiH20(3) GiH21(1) GiH22(1) GiH23(1) GiH24(1) GiH25(1) GiH26(1) GiH27(1) GiH28(1) |
|  | Italy | 3 | 0.667 | 0.00211 | GiH29(2) GiH30(1) |
|  | Poland | 11 | 0.873 | 0.00547 | GiH31(4) GiH32(1) GiH33(1) GiH34(1) GiH35(1) GiH36(2) GiH37(1) |
|  | DQ, China | 11 | 1.000 | 0.01349 | GiH13(1) GiH26(1) GiH38(1) GiH39(1) GiH40(1) GiH41(1) GiH42(1) GiH43(1) GiH44(1) GiH45(1) GiH46(1) |
|  | YL, China | 3 | 1.000 | 0.00633 | GiH20(1) GiH47(1) GiH48(1) |
| *G. nasalis* | KNR, China | 30 | 0.970 | 0.00818 | GnH1(2) GnH2(3) GnH3(1) GnH4(3) GnH5(1) GnH6(1) GnH7(2) GnH8(1) GnH9(1) GnH10(2) GnH11(3) GnH12(1) GnH13(1) GnH14(2) GnH15(1) GnH16(1) GnH17(1) GnH18(1) GnH19(1) GnH20(1) |
|  | DL, China | 24 | 0.902 | 0.00948 | GnH2(1) GnH6(4) GnH7(1) GnH12(4) GnH17(6) GnH21(1) GnH22(1) GnH23(1) GnH24(1) GnH25(1) GnH26(1) GnH27(1) GnH28(1) |
|  | MD, China | 9 | 0.500 | 0.00791 | GnH6(6) GnH17(3) |
|  | Italy | 3 | 1.000 | 0.01266 | GnH29(1) GnH30(1) GnH31(1) |
|  | Poland | 10 | 0.844 | 0.00587 | GnH32(3) GnH33(1) GnH34(1) GnH35(2) GnH36(3) |
| *G. nigricornis* | KNR, China | 29 | 0.953 | 0.01663 | GniH1(1) GniH2(2) GniH3(2) GniH4(2) GniH5(1) GniH6(5) GniH7(1) GniH8(1) GniH9(1) GniH10(3) GniH11(1) GniH12(3) GniH13(1) GniH14(1) GniH15(1) GniH16(1) GniH17(1) GniH18(1) |
|  | DL, China | 16 | 0.967 | 0.01665 | GniH7(1) GniH8(3) GniH19(1) GniH20(1) GniH21(1) GniH22(1) GniH23(1) GniH24(1) GniH25(1) GniH26(2) GniH27(1) GniH28(1) GniH29(1) |

**Table S2.** Number of individuals (*n*), haplotypes, number of haplotypes (denoted in parentheses), haplotype diversity (*Hd*), and nucleotide diversity (*π*) based on the mitochondrial cytochrome oxidase II gene in *G. pecorum*, *G. intestinalis*, *G. nasalis*, and *G. nigricornis*.

| Species | Location | *n* | *COII* gene | | |
| --- | --- | --- | --- | --- | --- |
|  |  |  | *Hd* | *π* | Haplotypes |
| *G. pecorum* | KNR, China | 50 | 0.917 | 0.00488 | GpH1(1) GpH2(2) GpH3(6) GpH4(10) GpH5(8) GpH6(1) GpH7(5) GpH8(1) GpH9(1) GpH10(1) GpH11(3) GpH12(1) GpH13(1) GpH14(1) GpH15(1) GpH16(1) GpH17(1) GpH18(1) GpH19(1) GpH20(1) GpH21(1) GpH22(1) |
|  | DL, China | 19 | 0.947 | 0.00699 | GpH2(1) GpH5(3) GpH17(3) GpH23(1) GpH24(1) GpH25(1) GpH26(1) GpH27(3) GpH28(1) GpH29(1) GpH30(1) GpH31(1) GpH32(1) |
|  | MD, China | 28 | 0.942 | 0.02035 | GpH2(2) GpH3(1) GpH4(2) GpH10(1) GpH33(2) GpH34(6) GpH35(1) GpH36(3) GpH37(1) GpH38(1) GpH39(2) GpH40(1) GpH41(1) GpH42(1) GpH43(1) GpH44(1) GpH45(1) |
| *G. intestinalis* | KNR, China | 17 | 0.949 | 0.00746 | GiH1(2) GiH2(1) GiH3(1) GiH4(1) GiH5(1) GiH6(4) GiH7(1) GiH8(1) GiH9(1) GiH10(1) GiH11(1) GiH12(1) GiH13(1) |
|  | DL, China | 25 | 0.960 | 0.00507 | GiH6(4) GiH9(3) GiH14(1) GiH15(3) GiH16(1) GiH17(1) GiH18(1) GiH19(1) GiH20(1) GiH21(1) GiH22(1) GiH23(1) GiH24(1) GiH25(1) GiH26(1) GiH27(1) GiH28(1) GiH29(1) |
| *G. nasalis* | KNR, China | 30 | 0.945 | 0.00825 | GnH1(2) GnH2(4) GnH3(1) GnH4(3) GnH5(1) GnH6(1) GnH7(2) GnH8(1) GnH9(2) GnH10(2) GnH11(5) GnH12(1) GnH13(2) GnH14(1) GnH15(1) GnH16(1) |
|  | DL, China | 24 | 0.931 | 0.00898 | GnH5(1) GnH6(1) GnH11(5) GnH17(1) GnH18(2) GnH19(2) GnH20(1) GnH21(4) GnH22(1) GnH23(1) GnH24(1) GnH25(2) GnH26(1) GnH27(1) |
|  | MD, China | 9 | 0.889 | 0.00969 | GnH27(2) GnH28(2) GnH29(2) GnH30(2) GnH31(1) |
| *G. nigricornis* | KNR, China | 29 | 0.901 | 0.01220 | GniH1(2) GniH2(8) GniH3(2) GniH4(2) GniH5(2) GniH6(2) GniH7(2) GniH8(3) GniH9(1) GniH10(3) GniH11(1) GniH12(1) |
|  | DL, China | 16 | 0.942 | 0.01096 | GniH2(4) GniH6(1) GniH13(1) GniH14(1) GniH15(1) GniH16(1) GniH17(1) GniH18(1) GniH19(1) GniH20(2) GniH21(1) GniH22(1) |

**Table S3.** Neutrality test for *G. pecorum*, *G. intestinalis*, *G. nasalis*, and *G. nigricornis* in different geographical locations.

| location | *G. pecorum* | | *G. intestinalis* | | *G. nasalis* | | *G. nigricornis* | |
| --- | --- | --- | --- | --- | --- | --- | --- | --- |
|  | Tajima’s *D* | Fu’s *Fs* | Tajima’s *D* | Fu’s *Fs* | Tajima’s *D* | Fu’s *Fs* | Tajima’s *D* | Fu’s *Fs* |
| ***COI*** |  |  |  |  |  |  |  |  |
| KNR | −1.7114* | −9.0983** | −1.0289 | −5.1901* | −1.0597 | −9.3466** | 0.3204 | −2.2922 |
| DL | −1.4293 | −5.4245* | −0.9077 | −9.1242** | −0.3888 | −1.6058 | 0.2072 | −2.5482 |
| MD | 0.9328 | 1.7895 | − | − | 1.6709 | 7.1593 | − | − |
| DQ | − | − | −1.3361 | −4.7508** | − | − | − | − |
| YL | − | − | 0.0000 | 0.1335 | − | − | − | − |
| Italy | − | − | 0.0000 | 1.0609 | 0.0000 | 0.9008 | − | − |
| Poland | − | − | 0.0503 | −1.2875 | −0.2041 | 0.8229 | − | − |
| ***COII*** |  |  |  |  |  |  |  |  |
| KNR | −1.9220** | −11.5392** | −1.7969* | −4.9907* | −0.6003 | −3.5773 | 0.9386 | 1.0321 |
| DL | −1.4877* | −4.3579* | −1.4648 | −12.2528** | −0.2853 | −2.6224 | −0.1538 | −2.4636 |
| MD | 1.0004 | −0.5060 | − | − | 1.0034 | 1.9196 | − | − |

**P* < 0.05, ***P* < 0.01.

**Table S4.** Maximum likelihood estimates of population size (*θ*), exponential growth rate (*g*), and migration rate for different populations of *G. pecorum*, *G. intestinalis*, *G. nasalis*, and *G. nigricornis*.

| Population(*i*) | θ | *g* | Migration rates | | | | | | |
| --- | --- | --- | --- | --- | --- | --- | --- | --- | --- |
|  |  |  | KNR→*i* | DL→*i* | MD→*i* | Italy→*i* | Poland→*i* | DQ→*i* | YL→*i* |
| Gp-KNR | 0.0302 | 169.1 | − | 366.40 | 13.79 | − | − | − | − |
| Gp-DL | 0.0400 | 517.6 | 4.05 × 10^−5^ | − | 49.41 | − | − | − | − |
| Gp-MD | 0.0124 | −1048.1 | 357.80 | 38.69 | − | − | − | − | − |
| Gi-KNR | 0.0833 | 1883.0 | − | 512.85 | − | 219.79 | 2.92 × 10^−4^ | 219.79 | 73.26 |
| Gi-DL | 0.0311 | 1410.4 | 1.43 × 10^−4^ | − | − | 715.51 | 1.40 × 10^−4^ | 268.27 | 44.75 |
| Gi-Italy | 0.0138 | 1344.0 | 2.09 × 10^−4^ | 2.09 × 10^−4^ | − | − | 134.48 | 1.36 × 10^−4^ | 2.10 × 10^−4^ |
| Gi-Poland | 0.0121 | 472.1 | 1.33 × 10^−4^ | 1.32 × 10^−4^ | − | 1.33 × 10^−4^ | − | 1.33 × 10^−4^ | 1.61 × 10^−4^ |
| Gi-DQ | 4.6511 | 3093.4 | 1.31 × 10^−4^ | 132.07 | − | 99.05 | 1.24 × 10^−4^ | − | 165.09 |
| Gi-YL | 0.0074 | 909.8 | 6.61 × 10^−4^ | 2961.70 | − | 6.41 × 10^−4^ | 6.19 × 10^−4^ | 6.31 × 10^−4^ | − |
| Gn-KNR | 0.0482 | 1634.2 | − | 877.21 | 250.75 | 41.77 | 1.53 × 10^−4^ | − | − |
| Gn-DL | 0.0229 | 601.6 | 374.49 | − | 436.96 | 1.04 × 10^−4^ | 9.59 × 10^−5^ | − | − |
| Gn-MD | 0.0008 | 1955.1 | 5.93 × 10^−4^ | 665.43 | − | 5.81 × 10^−4^ | 5.83 × 10^−4^ | − | − |
| Gn-Italy | 0.2883 | 639.3 | 1.16 × 10^−4^ | 1.59 × 10^−4^ | 1.51 × 10^−4^ | − | 1.32 × 10^−4^ | − | − |
| Gn-Poland | 0.0246 | 1123.4 | 41.57 | 1.37 × 10^−4^ | 2.24 × 10^−4^ | 41.57 | − | − | − |
| Gni-KNR | 0.0244 | 162.3 | − | 456.95 | − | − | − | − | − |
| Gni-DL | 0.0200 | 282.2 | 476.88 | − | − | − | − | − | − |

*Abbreviations*: *Gi G. intestinalis*, *Gn* *G. nasalis*, *Gni* *G. nigricornis*, *Gp* *G. pecorum*
